# Increasing the Utility of Real-World Data to Inform Public Health Decision Making Through a US-based Private–Public Partnership: 10 Lessons Learned from a Principled Approach to Rapid Pandemic RWE Generation

**DOI:** 10.1007/s43441-025-00748-4

**Published:** 2025-03-18

**Authors:** Nicolle M. Gatto, Elizabeth M. Garry, Melanie Wang, Névine Zariffa, Laura Roe, Aloka Chakravarty, Donna Rivera

**Affiliations:** 1 Aetion, Inc, 5 Pennsylvania Plaza, New York, NY 10001 USA; 2 NMD Group Inc, Bala Cynwyd, PA USA; 3 MMCi, Verily, San Francisco, CA USA; 4 U.S. Food and Drug Administration, Silver Spring, MD USA

**Keywords:** COVID-19, Epidemiologic methods, Pandemic preparedness, Real-world data, Real-world evidence, SARS-CoV-2

## Abstract

In response to the COVID-19 pandemic, a collaborative public–private partnership was launched to harness evidence from rapidly accruing real-world data (RWD) in various healthcare settings, with the goal of characterizing and understanding COVID-19 in near real-time, by applying rigorous epidemiological methods and defining research best practices. Projects were conducted in 4 phases: Research Planning and Prioritization, Protocol Development, Protocol Implementation, and Results Dissemination. During these projects, areas were identified with a current or future need to enhance existing best practices. This report provides a summary of our research processes, including application of new and existing practices, along with key learnings related to the challenges of conducting research when the clinical landscape is rapidly evolving as was the case during the first year of the COVID-19 pandemic. Such processes and learnings may be helpful to the broader research community when using RWD to understand or address future public health priorities.

**Supplementary Information:**

The online version contains supplementary material available at 10.1007/s43441-025-00748-4.

## Introduction

Researchers generating real-world evidence (RWE) for COVID-19 epidemiology and treatments faced unparalleled challenges as they accessed real-world data (RWD) sources, due to quickly evolving infection patterns and associated clinical care. The global pandemic demanded real-time analyses, continuous evaluation of new and emerging sources of data, and scrutiny of missing data elements or inconsistent patterns that had the potential to be informative. While data feasibility and selection considerations (e.g., data lag) for observational studies are not new, the novel coronavirus (COVID-19) further illustrated the need for fit-for-purpose data assessment with a deep understanding of data sources. The rapidly changing treatment paradigm also required agile adaptability of study designs and methods. Authorities often had to make decisions based on the best available evidence. As a result, collaborative public–private partnerships, such as the Reagan-Udall Evidence Accelerator, were quickly assembled, ultimately yielding important COVID-19 clinical and epidemiologic insights (for example, see [1–7]), as well as process and methods recommendations (for example, see [8–11]) that may be applied beyond COVID-19 to future pandemics or other public health crises.

As part of additional public health response efforts to understand COVID-19 by harnessing available real world data sources, the US Food and Drug Administration (FDA) established a research collaboration with Aetion, Inc. in May 2020. To answer research questions quickly, the collaborative team set out to identify and analyze select US data sources to characterize COVID-19 patient populations, contribute to the scientific evaluation of potential therapeutic interventions, and apply efficient approaches such as using an analytic platform for multiple research questions. The fundamental components of this project were to leverage and understand the features of various diverse data sources, utilize good research practices and methodologies, and evaluate operational processes that could enable iterative improvement for subsequent investigations. The applied research practices, learnings, and newly developed tools from this collaboration may be useful to investigators conducting RWD-based research beyond COVID-19, particularly studies employing accelerated analytics to support timely decision-making for other public health priorities. These accelerated RWE generation practices and learnings are described below and summarized in Table 1.

**Table 1:** Study steps, practices and learnings

Phase 1: Research planning and prioritization	
Purpose: Enable efficiency, timely and meaningful evidence	
Study step	Practice details
Generate timely evidence	● Applied platform approach enhanced speed of analysis and efficiency, while providing transparent reporting ● Pre-identified relevant data sources with short data lag and frequent refreshes and available to be ingested into a platform, to serve as “universe” of data sources for study specific feasibility assessments ● Refreshed data source(s) as new data became available (in most cases bi-weekly); *for efficiency, in future would refresh at prespecified study points (Key Lesson #1)*
Systematically evaluate and characterize each data source of interest	● Gathered data dictionaries and/or other documentation (when available) ● Met with data vendors/providers to understand essential information such as data provenance and completeness of fields needed for most research questions (e.g., age) ● Compiled key information for each data source and organized data characterization into newly developed standard template (Table 2) to support feasibility assessment; *for efficiency, in future would share the standard template with data vendors/providers and ask them to complete it (and/or update it if relevant; Key Lesson #2)*
Define initial research questions	● Defined preliminary research questions of interest with collaboration members ● Allowed for research question iteration and reprioritization as needs changed and/or based on observed treatment pattern changes and learnings from other research; *flexibility to pivot on downstream research questions was important (Key Lesson #3)*

Phase 2: Protocol development	
Purpose: Enable meaningful, valid and transparent evidence	
Study step	Practice details
Prespecify research question(s) and objective(s)	● Articulated detailed research question and objectives to ensure core study elements were identifiable; *doing so ensured laser focus for data source evaluation (Key Lesson #4)*
Initiate real-world study design and identify fit-for-purpose data	● Used existing frameworks and templates to: ○ Guide design choices and rationale, and identify minimal criteria for real-world data to meet the needs of the study [14, 26, 27] ○ Illustrate initial study design and key assessment windows (e.g., baseline, exposure, follow-up) [37] ○ Identify potential confounders (comparative studies only) [28, 29] ○ Identify fit-for-purpose data source(s) (i.e., reliable and relevant) to meet the needs of a specific research question [15] ○ *For efficiency, in future would use newly published template with combined study design and data fitness assessment steps* [34] *(Key Lesson #5)*
Explore selected data source(s)	● While carefully avoiding connecting treatments and outcome of interest (e.g., outcome blinded,) explored data and documented findings to maintain objectivity, but allowed evidence based protocol decisions such as: ○ Completeness and general trends of key variables (e.g., inclusion/ exclusion criteria, subgroups, primary outcome) ○ Considered alternate definitions/time windows for key variables (e.g., confirmed COVID-19 or mortality) ○ Compared general trends of key variables to internal and external benchmarks (as available) to inform final variable definitions and time windows ○ Identified potential threats to validity that warrant analysis considerations, should be further explored via planned sensitivity analyses, and/or should be documented as potential limitations of the data source ○ *Data explorations, maintaining blinding, were necessary to yield valid and interpretable study findings. In future, would consider also applying newly published process that incorporates diagnostic steps as a standard part of protocol design *[36]* (Key Lesson #6)*
Develop, finalize, and post protocol	● Followed comprehensive protocol development standards [23–25]; for efficiency, in future would consider using newly published protocol template that harmonizes protocol development best practices and guidance and incorporates templated study analytic details [16, 39] ● Included a final visual study design and timing diagram [37] ● Included a diagnostic implementation step with objective criteria / checklists for moving from diagnostics to inferential analyses ● Planned contingent analyses and/or sensitivity analyses (with rationale) to address remaining validity concerns and/or to evaluate alternate design choices ● Incorporated phased study implementation into the protocol design to help us remain blinded to treatment-outcome associations (to maintain objectivity) during the design and baseline diagnostic phases of the comparative study; *For validity, in the future we would (Key Lesson #7):* ○ *Reanalyze treatment pattern analyses prior to beginning the inferential phase* ○ *Add prespecified analytic contingencies corresponding to any failed diagnostic criteria/thresholds, triggering adjustment for informative censoring if warranted* ○ *Add a second phase diagnostic step that allow evaluation of post-baseline treatment patterns, crossover, and reasons for censoring, prior to beginning the inferential phase but only to be implemented after the first (baseline) diagnostic phase is complete (*Fig. 1*)* ○ *Specify objective diagnostic criteria for post-baseline treatment changes to identify potential informative censoring due to shifts in the treatment paradigm* ● For the comparative study, posted final protocol on a publicly available website (here, clinicaltrials.gov) prior to implementation

Phase 3: Protocol implementation	
Purpose: Enable meaningful, valid and transparent evidence	
Study step	Practice details
Conduct diagnostic analyses and check criteria	● Conducted prespecified diagnostics ● If study diagnostics were not met initially, planned contingencies and iterations were implemented until all criteria could be satisfied ● Study diagnostic findings were reviewed by all key collaboration members, and findings and decisions were recorded; *recording diagnostic findings and decisions further enabled transparency and efficient presentation and publication (Key Lesson #8)*
Conduct descriptive analyses (and inferential, if relevant)	● After study diagnostics were met and agreed upon with the team, any remaining descriptive analyses were implemented, followed by inferential analyses if applicable ● Implementation findings and decisions, along with the rationale were documented
Synthesize and interpret results	● Relevant post hoc analyses were identified after all planned protocol analyses were complete, reviewed for quality control, synthesized and reviewed by all collaboration members; *allowed focused identification of key additional analyses to further aid in the interpretation of results and enabled team alignment on analytical steps (Key Lesson #9)* ● Documented post hoc analysis plans (as a separate ‘post hoc analytic plan’) prior to implementation

PHASE 4: RESULTS DISSEMINATION	
Purpose: Enable transparent and trustworthy evidence	
Study step	Practice details
Disseminate findings	● Final results were shared publicly through peer reviewed conference abstracts and presentations, in FDA Science Forums and publications, where feasible; *Use of “brief report” style rather than full length manuscripts might be valuable to further accelerate dissemination (Key Lesson #10)*

Italicized text represents key lessons

## Evidence Generation Process

At the start of this research collaboration, a multidisciplinary team of clinicians, epidemiologists, biostatisticians, data experts, and project managers was formed. The collaborative research team established systematic processes designed to generate meaningful, valid, expedited, and transparent RWE [12, 13], leveraging existing recommendations and collective experience, and developing novel approaches when needed. Our research process consisted of 4 phases: (1) research planning and prioritization, (2) protocol development, (3) protocol implementation, and (4) results dissemination, with operational steps underlying each phase. In Phase 1, we developed a new template to facilitate data source characterization. In Phases 2 and 3, we applied existing study-specific tools (e.g., templates, decision trees, step-by-step processes) to enable decision-grade RWE during study design [14], fit-for-purpose data assessment [15], protocol finalization [14, 16], and protocol implementation [16]. Additionally, through Phase 2, we developed a new study phase diagram to enable communication and transparency of our protocol implementation process.

### Phase 1: Research Planning and Prioritization

#### Generate Timely Evidence

The global pandemic necessitated rapid answers to pressing research questions. To allow the research team to implement multiple studies to generate timely evidence, this collaboration used a platform approach to enhance the efficiency, and therefore speed, of analytic implementation. Generally, a platform approach involves use of data with: (a) clear and appropriate data transformations; (b) validated analytic workflows with documentation; (c) reproducible analyses, rather than traditional line-coding; and (d) direct access to underlying assumptions and analytic measures to enable timely evidence generation. Aetion® Substantiate [17] was able to reuse previously built algorithms for defining variables of importance for COVID-related research and the data (where prespecified in protocols) for multiple studies. It also facilitated transparent reporting of all variable algorithms and analytic steps.

Given the rapidly changing clinical care of and epidemiology of COVID-19, the team sought data sources with multiple linked or linkable data types, short lag times (e.g., 1 month lag in time between data capture and analysis ready data,) and frequent refreshes available (e.g., refreshed every 2–4 weeks to add the most recent data), that could be rapidly connected to and analyzed on the analytic platform. Further, the ability to accelerate the contracting process and delivery of the initial data was also considered. While the goal was to provide accurate evidence as quickly as possible, timelines for each study varied considerably due to numerous factors (e.g., complexity, sample size requirements, and number of parallel studies at a given time).

##### Key Lesson #1: Strategic Data Refreshing

For the data source [20] that was continually refreshed, new data became available generally every two weeks. While data with a short lag time was important for ensuring meaningful evidence—for data explorations, diagnostics, and analyses—refreshing at specific study points (e.g., just before starting the Protocol Implementation) may save effort and time. However, to maintain objectivity, data should only be refreshed prior to study initiation and per protocol.

#### Systematically Evaluate and Characterize Each Data Source of Interest

While there were many sources of data being assembled to support COVID-19 research, seven US sources were identified as potential candidates within this project. As a first step, data dictionaries and other written documentation were reviewed (if available). The team then confirmed understanding of essential information such as data provenance and completeness of fields needed for most of the potential research questions (e.g., age, sex) with data providers. For each data source, these elements were compiled into written summaries and organized into a table to effectively communicate key dataset components (e.g., sample size, inclusion criteria, update frequency, type, and level of documentation on source data and transformation) for each data source (Table 2). Given the importance of near real-time data, regular and frequent communication with potential data partners was required to fully understand the composition and nuances of the RWD sources. As an initial step in the process, evaluation of the metadata was needed to assess study feasibility.

**Table 2:** Standard data characterization template

#	Component/Question	Response
	**Section 1: Overall Description**	
1	Data source description / any inclusion criteria applied (e.g., who is in the available data?)	
2	Date of most recent data cut	
3	Total number of unique people in the data	
4	Geographic Area (e.g., states covered)	
5	Number of data types	
6	Data types (e.g., insurance claims, hospital EHR, etc.)	
7	Data fields included in standard base dataset (i.e., without any special linkages)	
8	Summary of known missingness in the data overall and for key demographic fields (such as age, sex, race, state, etc.)	
9	Linkage potential across other datasets	
	**Section 2: Timeliness**	
10	Dates captured	
11	Data lag	
12	Refresh rate/frequency	
	**Section 3: Technical and Privacy**	
13	Data model (any relevant mapping/transformation)	
14	De-identification/privacy protection (including tokenization measures)	
15	Type + level of documentation on source data	
16	Type + level of documentation on data transformations	
17	What tools, environments, and processes are required to connect this data based on data privacy and security?	
18	Does the data include variables that map to coding systems (e.g., ICD-9, ICD-10, CPT/HCPCS, NDC)?	

##### Key Lesson #2: Establish Structured Data Characterization

Use of a standard data characterization form – to be completed and regularly updated by data providers – can increase efficiency and speed in this initial phase of work. An example template is shown in Table 2. The intent is to minimize the burden on the data providers prior to contracting, while including enough information to evaluate initial aspects of the data that inform data relevance and reliability [18] and whether the data are appropriate for inclusion in the data feasibility assessment for a specific research question.

#### Define Initial Research Questions

Preliminary research questions of interest were identified at the start of the research collaboration through regulatory research priorities, collective idea generation, and review of the relevant literature available. Many of the initial questions focused on characterizing COVID-19 natural history and treatment patterns (Table S1), or on the safety and effectiveness of early treatments (e.g., hydroxychloroquine). For each preliminary research question of interest, specific objectives were defined, and then the research questions were prioritized based on collaborative agreement of current clinical relevance and whether the research question represented a foundational aspect necessary for subsequent assessments (numbers in Table S1 are in order of planned initiation). Research questions were then revised and reprioritized as scientific needs changed over time based on observed treatment patterns (often following emergency use authorization changes) and emerging knowledge from implementation of prior collaboration research questions, other COVID-19 collaborative research efforts such as the COVID-19 Evidence Accelerator [19], and published research.

##### Key Lesson #3: Prioritize Initial Research Questions

Developing initial research questions allowed prioritization of foundational aspects that were used in subsequent assessments, accelerating implementation. However, flexibility to pivot on downstream research questions was important given learnings from the foundational work and the rapidly evolving health response.

### Phase 2: Protocol Development

Protocol development included four operational steps spanning from articulating a specific research question to finalizing the protocol. Protocol development included a step to systematically identify fit-for-purpose data for the intended research question, as well as a step allowing descriptive exploration of the selected data prior to study design and protocol finalization. For full transparency and replicability, all study details, including key study design parameters and operational and implementation details, were recorded using structured templates [14–16].

#### Articulate Research Question(s) and Objective(s)

Within the first year of the collaboration, the research team addressed 10 research questions – 9 descriptive and 1 comparative – leveraging RWD to better understand COVID-19, specifically the natural history, potential therapies, and diagnostics (see Table S1) within two US administrative healthcare data sources that comprised claims, hospital chargemaster and electronic health records [20–22]. For each study, the research question and objectives were prospectively developed to be sufficiently detailed to ensure alignment among the team and to clearly specify the population and key subgroups of interest, treatment(s), outcome(s), covariates of interest, and timeframe [13, 23, 24].

##### Key Lesson #4: Prespecify Research Question and Objectives

Articulating the detailed research question in a structured and comprehensive manner using an existing framework and template [14], facilitated alignment with the research team to ensure laser focus for data source evaluation.

#### Initiate Real-World Study Design and Identify Fit-For-Purpose Data

For each study, existing frameworks and templates were used to guide design choices, capture the rationale, and identify minimal criteria for a RWD source to meet the needs of the study [12, 14–16]. Study design diagrams illustrating key assessment windows (e.g., baseline, exposure, follow-up) were used for all studies to enable study team decision-making and reproducibility. Additionally, for the comparative study [25] the team designed the study as an emulation of a hypothetical pragmatic trial [26, 27] and used Directed Acyclic Graphs [28, 29] to identify potential confounders (care setting, data types, completeness of key data fields, numbers of patients).

Once the minimal data criteria were defined for each study, a data feasibility framework and structured templates were used to guide the data selection process [15]. For data to be fit-for-purpose, data must be both reliable and relevant [30–32]. Reliability broadly relates to the accuracy of the data [31, 32], and data relevance pertains to the availability of sufficient patients with key study data elements and representativeness [30, 33]. If a fit-for-purpose data source could not be identified, the research question was revised or not pursued.

##### Key Lesson #5: Streamline Templates for Study Design and Data Decision Rationale

While the approach for identifying fit-for-purpose data was considered successful as an adoptable model for future studies, the team felt relevant components of existing reporting templates and required fields [14–16] could be merged into one source from the onset as a “master” approach for decision-making. Based in part on this experience, a combined template for study design and data fitness assessment plus specific references to the analytic detail has since been published that may be used to reduce duplication and produce more transparent documentation [34].

#### Explore Selected Data Source(s)

Once the data for a particular research question were accessible (i.e., received, processed, and available on the platform), data were explored to inform protocol decisions on variable definitions and time windows (e.g., the length of the pre-hospitalization baseline period), and identify additional potential threats to validity (e.g., possible misclassification of oxygen support among the group identified as not receiving any supplemental oxygen) and the need for protocol-specified sensitivity analyses to evaluate design decisions and robustness of findings. To maintain objectivity in the design and analysis of each research question, planned data explorations were documented, and any treatment-outcome associations remained blinded per Good Pharmacoepidemiology Practice [35] (e.g., treatments and outcomes were not linked, and treatment-outcome associations were not analyzed or viewed to ensure independence of study design decisions).

The team evaluated and documented the completeness of key variables necessary for defining the minimal criteria such as inclusion/exclusion criteria, subgroups, as well as various algorithms for defining the primary outcome (again, without “unblinding” treatment-outcome associations) to verify data fitness for purpose. General trends of key variables were compared with internal and external benchmarks (as available in existing literature or nationally reported government public health statistics). This proved an important step that highlighted data nuances essential to maximizing validity for each research question/dataset*.* For example, in the comparative study, this exercise guided the decision to truncate the cohort entry date selection period to end 60 days earlier than the end of the near-real-time data to ensure complete data for reporting the mortality endpoint [25]. The detailed feasibility assessment was refined as data explorations uncovered subtle variations and insights. As during the data feasibility assessment, communication with data partners was required to fully understand the underlying features of the data (e.g., data provenance, data linkage, data transformation), make decisions around adapting variable definitions and the study design, and to ensure transparent acknowledgment of potential limitations.

Once completed, the findings were documented, and all variable definitions or algorithms (how all variables were defined and operationalized in the study), and assessment windows were documented in the structured template [16], as an appendix to the final protocol (see for example, Appendix A in the posted protocol [25]). Key study design decisions derived from the exploratory step were reviewed by all collaborators and incorporated into a final protocol with consensus.

##### Key Lesson #6: Feasibility Assessment Confirmation Through Data Exploration

Data explorations, maintaining blinding, were necessary to yield valid and interpretable study findings. For future studies a newly published process that incorporates diagnostic steps as a standard part of protocol design is available [36].

#### Develop, Finalize, and Post Protocol

Protocol Development incorporated the key design decisions derived during the exploratory step and concluded with protocol completion. Following published protocol standards and regulatory guidances [35], all study design details were documented in protocols. Protocols included descriptions of the RWD source and fit-for-purpose rationale; study design details, including assessment windows for baseline, exposure, and follow-up (including censoring criteria) [37]; and a statistical analysis plan including primary, secondary, and planned sensitivity analyses. Once protocols were developed for initial research questions, there was an appreciable efficiency gain from using the protocol template and relevant protocol text (e.g., when applying to the same data source, approach and/or study parameters). Visual study design diagrams [37] with assessment windows were also included [25, 38]. For the comparative study, graphical depictions of potential confounders and potential threats to internal validity (e.g., misclassification, incomplete data, and residual confounding) were proactively identified, and the protocol specified how these limitations would be addressed in the study design and analysis.

Protocols specified phased implementation with (1) built-in diagnostic tests and objective criteria to be met before conducting the analyses (e.g., specific requirements for baseline covariate balance), (2) analytic contingencies tied to each diagnostic test, and (3) required team consensus to move to each subsequent implementation phase [25]. Similar to the data exploration step, these protocolized diagnostics and criteria were carefully specified to avoid unblinding any treatment-outcome association to maintain objectivity (i.e., to ensure that implementation decisions were unbiased). We developed a protocol template aligned with published standards and guidance, enabling efficiency and consistency in our documentation. However, there is now a protocol template designed to harmonize protocol development best practices and guidance, and incorporate templated study analytic details [16, 37, 39] that were not available for consideration in this study.

Existing guidelines unanimously agree on the importance of posting final protocols for non-interventional (observational) studies. Posting final protocols for such observational studies on a publicly available website (such as ClinicalTrials.gov or ENCePP.eu) prior to study implementation provides transparency. Public posting is one way that might provide external confidence in the prespecified design and allows evaluation of whether the implementation was influenced by the findings [32], integral for studies intended to support causal inferences [40]. Therefore, the final approved protocol for the comparative study was publicly posted on ClinicalTrials.gov [25]. All studies were also covered under an IRB exemption determination from New England IRB.

##### Key Lesson #7: Protocol Design

**Figure 1:**
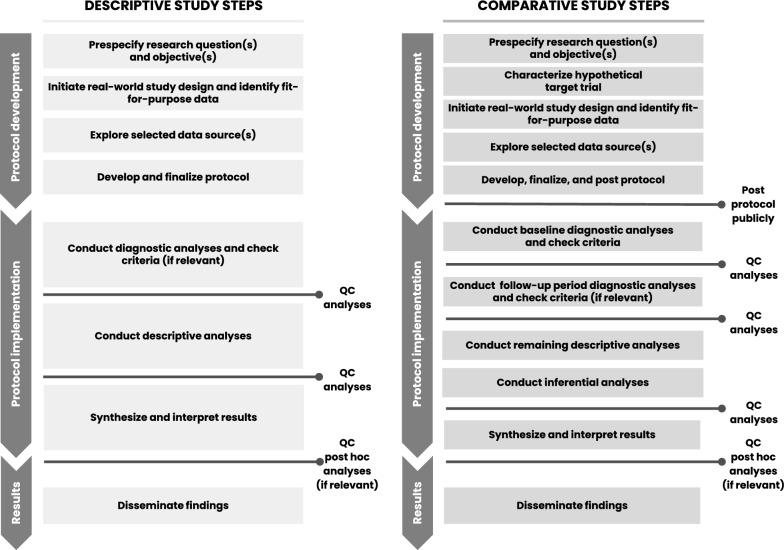
Proposed process for descriptive and comparative effectiveness studies with designated checkpoints required to proceed.

- Incorporation of phased study conduct into the protocol design helped us remain blinded to treatment-outcome associations (to maintain objectivity) during the design and baseline diagnostic phases of the comparative study. However, there are three interrelated aspects that could be incorporated into future studies, particularly when clinical care for the disease or condition of interest is rapidly evolving.
- **Treatment Patterns:** Treatment pattern findings from the exploration phase did not signify the potential for post-baseline treatment changes, and therefore adjustment for potential informative censoring was not included in the comparative protocol. However, treatment patterns drastically changed over the period from Protocol Development to Protocol Implementation. These changes, combined with our strict adherence to the protocolized phased approach (which was specified to ensure objectivity), created challenges in the comparative study. Because evaluation of post-baseline data until the inferential phase was not allowed, the substantial treatment crossover that occurred just after the study index date was not known and it was not feasible to amend the protocol prior to the inferential phase. Thus, inverse probability of censoring weighting was used to evaluate the impact of this crossover on the hazard ratios post hoc [41]. The need to reanalyze changing treatment patterns prior to beginning the inferential phase was a lesson learned through this research.
- **Analytic Contingencies:** As noted above, due to the drastic treatment changes, some design and analysis decisions made based on initial data explorations were not fully aligned to the actual study data. To address this in future work, we would add objective diagnostic criteria for post-baseline treatment changes to identify potential informative shifts in the treatment paradigm prior to the inferential phase. Analytic contingencies would be prespecified that correspond to any suboptimal diagnostic criteria triggering adjustment for informative censoring, if warranted. Although it is difficult to anticipate every possible change in patient care or the research environment, it is crucial we build in analytic contingencies to address major changes in clinical care (such as potential channeling towards or away from study treatments over time) that would otherwise impact target trial emulation [42, 43]. Prespecified contingency plans may also allow revision of the research question if the study is likely to have reduced interpretability or relevance due to changes in standards of care.
- **Diagnostic Criteria:** To evaluate and analyze informative treatment changes in prespecified analyses, objective diagnostic criteria are needed for post-baseline treatment changes. Future comparative study protocols may consider a second phase diagnostic step that could include evaluation of post-baseline treatment patterns, crossover, and reasons for censoring. Figure 1 provides a proposed phased study process that incorporates the two diagnostic phases for comparative studies—one for baseline data diagnostics and a second for follow-up period diagnostics. However, the analysis of post-baseline data requires careful consideration to ensure outcome blinding of treatment-specific endpoints to support decision making. Thus, this second diagnostic phase would be implemented only after the first (baseline) diagnostic phase is complete to ensure implementation objectivity. Like the first phase diagnostics, the protocol should specify objective criteria to be met before initiating inferential analyses and/or should link diagnostics findings to specific contingent analyses. Having this second diagnostic phase would also allow the research team to consider amending the protocol prior to inferential implementation, if warranted by an unanticipated change that would otherwise reduce the interpretability of the study findings. In fact, these learnings were applied and further developed in a methodologically focused evaluative study [44, 45].

### Phase 3: Protocol Implementation

#### Conduct Diagnostic Analyses and Check Criteria

For all research questions, we implemented protocols as planned, starting with the prespecified diagnostic step. If study diagnostic criteria were not met initially, planned contingencies and iterations were implemented until all criteria could be satisfied. This included waiting for data refreshes if a sample size criterion was not met or consolidating or removing parameters of the propensity score model to satisfy the positivity assumption. Study diagnostic findings were reviewed by all key collaboration members, and findings and decisions were recorded [16].

##### Key Lesson #8: Prespecified Analysis Implementation

Evaluating diagnostic findings and recording decisions further enabled transparency and efficient presentation and publication, and minimized the number of post hoc analyses conducted.

#### Conduct Descriptive Analyses (and Inferential, if Relevant)

After study diagnostic criteria were reviewed with consensus from the team, any remaining descriptive analyses were implemented, followed by inferential analyses. Implementation findings and decisions, along with the rationale, were documented [16].

#### Synthesize and Interpret Results

Once all planned protocol analyses were completed and quality control processes were applied, data were synthesized and reviewed by all collaboration members. Post hoc analyses were identified by consensus and documented in the structured template [16]. Post hoc analytic plans were then created and implemented. Of note, these analyses may not be appropriate for decision making and may have limited interpretability.

##### Key Lesson #9: Post Hoc Analysis Implementation

Considering post hoc analyses only after all prespecified analyses were complete and reviewed allowed focused identification of key additional analyses to further aid in the interpretation of primary prespecified results. Documenting the objective and potential interpretation of post hoc analyses prior to implementation provided clear rationale to justify additional analyses and how they diverged from the prespecified analyses, and further enabled team alignment on these additional analytic steps.

### Phase 4: Results Dissemination

#### Disseminate Findings

For all treatment-specific research questions, final results were shared publicly through peer-reviewed conference abstracts and presentations (n = 7), in FDA Science Forums (n = 3), and as manuscripts (n = 5) (Table S1). Presentations and publications focused on prespecified analyses, and clearly delineated all post hoc findings when presented. The dissemination of these efforts supported understanding of treatment-related COVID-19 research, methodological approaches to ongoing regulatory science research efforts across centers to answer public health questions, as well as informing the broader clinical and scientific community.

##### Key Lesson #10: Peer-review Publication

While results were shared as quickly as was feasible and conference submissions were an efficient way to disseminate results relatively rapidly, manuscript submissions to journals presented challenges, as clinical and epidemiologic journals were inundated with COVID-19 submissions after the first year of the pandemic. Use of “brief report” style papers rather than full length manuscripts covering all primary and secondary analyses may have resulted in more timely manuscript peer review and publication. Additionally, exploring systems to better accommodate the need for peer review or provide a mechanism to incentivize such review to meet necessary public health challenges is worth evaluation.

## Conclusion

Deciphering COVID-19 data necessitated out-of-the-box nimble approaches and transparent research frameworks. Research groups worldwide were galvanized to understand COVID-19 leading to the formation of academic consortiums [46]; and non-profit organizations and industry collaborations to answer urgent treatment questions [38]. Collectively, the epidemiology community stepped up to use real-world data for a variety of purposes to address this urgent need, reflecting on lessons learned to prepare and act swiftly for another pandemic or public health priority. Several publications have since provided methodological considerations for evaluating COVID-19 treatments [9, 32, 47, 48].

Among others, this collaborative public–private partnership was launched to harness evidence from rapidly accruing data sources and apply rigorous epidemiological methods in response to the urgent need to characterize and understand COVID-19 in near real-time. We found that challenges related to conducting research when the clinical landscape is rapidly evolving, especially during the first COVID-19 pandemic year, were important to anticipate and challenging to address. While our applied good research practices were successful, the collaborative research team identified learnings at several steps. Two new tools were developed that may be useful to other researchers: 1) a standard template to capture meta-data (directly from data owners whenever possible), and 2) a phased study design and implementation diagram for descriptive and comparative studies that incorporates two study implementation diagnostic phases with clear diagnostic criteria with prespecified analytic contingencies to allow for relevant data-driven, and objective, implementation decisions. Taken together, our practices and learnings may enable future research collaborations established to generate accelerated RWE analytics and insights to make meaningful contributions necessary for evidence generation to support timely decision-making for other public health priorities.

## Supplementary Information

Below is the link to the electronic supplementary material.

Supplementary file1 (DOCX 25 KB)

Supplementary file2 (DOCX 16 KB)

## Data Availability

No datasets were generated or analysed during the current study.
