# Supplementary material for: Increasing the Utility of Real-World Data to Inform Public Health Decision Making Through a US-based Private–Public Partnership: 10 Lessons Learned from a Principled Approach to Rapid Pandemic RWE Generation
Source: Ther Innov Regul Sci. 2025 Mar 18;59(3):629–41. doi: 10.1007/s43441-025-00748-4 (PMC12018611; doi:10.1007/s43441-025-00748-4)
Supplement: Supplementary file 1 — Supplementary file1 (DOCX 25 KB) [file 43441_2025_748_MOESM1_ESM.docx]

**Table S1. Research Question Summary^1^**

| **#** | **Initial Research Question** | **Final Research Question** | **Reference(s)** |
| --- | --- | --- | --- |
| 1 | What are typical treatment patterns, co-medications, and effects on select health outcomes (safety/effectiveness) among patients with COVID-19 infection and treated with HCQ in an outpatient setting? | Among patients with COVID-19 infection and treated with Hydroxychloroquine (HCQ) in an outpatient setting, what are typical treatment patterns, co-medications, and effects on select health outcomes (safety/effectiveness)? | Evaluation only |
| 2 | Among patients with COVID-19 infection and treated with HCQ in an inpatient setting, what are typical treatment patterns, co-medications, and effects on select health outcomes (safety/effectiveness)?^2^ | | Stewart M, Rodriguez-Watson C, Albayrak A, et al. **COVID-19 Evidence Accelerator: A parallel analysis to describe the use of Hydroxychloroquine with or without Azithromycin among hospitalized COVID-19 patients.** PLoS One. 2021;16(3):e0248128.  Full-text available from: <https://www.ncbi.nlm.nih.gov/pmc/articles/PMC7968637/> |
| 3 | What is the landscape of COVID-19 care? | What is the landscape of COVID-19 care? For patients with COVID-19, describe baseline characteristics overall, by care setting, calendar time, race/ethnicity, and insurance type. What is the natural history of COVID-19 overall, and by subgroups of interest? | Okobi MA, Prince P, Roe LM, Rivera DR, Quinto K, Leonard S, Garry EM, Wang M, Gatto NM. Temporal changes in the baseline patient characteristics of COVID-19 patients. 37th International Conference for Pharmacoepidemiology - All Access, Virtual. Poster, Aug 2021.  Okobi MA, Prince P, Roe LM, Rivera DR, Quinto K, Leonard S, Garry EM, Wang M, Gatto NM. **Temporal changes in the baseline patient characteristics of COVID-19 patients**. In: Abstracts of the 37th International Conference on Pharmacoepidemiology & Therapeutic Risk Management, Virtual, August 23, 2021. Pharmacoepidemiol Drug Saf. 2021 Aug;30 Suppl 1:351. doi: 10.1002/pds.5305. PMID: 34431155.  Abstracts available from: <https://onlinelibrary.wiley.com/doi/10.1002/pds.5305>. |
| 4 | What are typical treatment patterns, co-medications, and effects on select health outcomes (safety/effectiveness) among patients hospitalized for COVID-19 infection and treated with corticosteroids of interest (dexamethasone, methylprednisolone, prednisone, and hydrocortisone) in an inpatient setting? | Determine whether treatment with dexamethasone (DEX) within 21 of admission among US patients hospitalized with COVID-19 diagnosis or SARS-CoV-2 infection reduces the risk of inpatient mortality within 28 days, overall and stratified by COVID-19 severity subgroups. | Gatto N. Effect of Dexamethasone on Inpatient Mortality Among Hospitalized COVID-19 Patients [Internet]. clinicaltrials.gov; 2022 Dec [cited 2022 Feb 11]. Report No.: NCT04926571. Available from: <https://clinicaltrials.gov/ct2/show/NCT04926571>  Garry EM, Easthausen IJ, Vititoe SE, Weckstein AR, Chakravarty A, Lasky T, Bradley MC, Perez-Vilar S, Rivera DR, Quinto K, Zhang Di, Zhao Y, Rajpal A, Baglivo A, Rassen JA, Leonard S, Gatto NM. [Estimating real-world outcomes under a rapidly evolving treatment paradigm: Dexamethasone and inpatient mortality among US hospitalized COVID-19 patients](https://www.fda.gov/science-research/fda-science-forum/estimating-real-world-treatment-effects-under-rapidly-evolving-treatment-paradigm). The 2023 FDA Science Forum. Advancing Regulatory Science Through Innovation. Poster, Session 6: Medical Countermeasures, Infectious Disease and Pathogen Reduction Technologies, June 2023.  Garry EM, Easthausen IJ, Vititoe SE, Weckstein A, Chakravarty A, Lasky T, Bradley MC, Perez-Vilar S, Rivera DR, Quinto K, Zhang Di, Zhao Y, Rajpal A, Baglivo A, Rassen JA, Leonard S, Gatto NM. **Estimating real-world outcomes under a rapidly evolving treatment paradigm: Dexamethasone and inpatient mortality among US hospitalized COVID-19 patients.** In: ABSTRACTS of ICPE 2022, the 38th International Conference on Pharmacoepidemiology and Therapeutic Risk Management (ICPE), Copenhagen, Denmark, 26–28 August 2022. Pharmacoepidemiol Drug Saf. 2022 Sep;31 Suppl 2:24-25. doi: 10.1002/pds.5518. PMID: 36148859.  Abstracts available from: <https://onlinelibrary.wiley.com/doi/10.1002/pds.5518>.  Garry EM, Easthausen IJ, Vititoe SE, Weckstein A, Chakravarty A, Lasky T, Bradley MC, Perez-Vilar S, Rivera DR, Quinto K, Zhang Di, Zhao Y, Rajpal A, Baglivo A, Rassen JA, Leonard S, Gatto NM. **Estimating real-world outcomes under a rapidly evolving treatment paradigm: Dexamethasone and inpatient mortality among US hospitalized COVID-19 patients**. 38th International Conference for Pharmacoepidemiology, Copenhagen, Denmark. Podium, Aug 2022. |
| 5 | Using positive SARS-CoV-2 PCR as the gold standard, what is the sensitivity of subsequent antibody testing, overall and stratified by subgroups of interest?^2^ | Using positive SARS-CoV-2 PCR as the gold standard, what is the percent positive agreement (PPA) of subsequent antibody testing, overall and stratified by subgroups of interest?^2^ | Rodriguez-Watson CV, Sheils NE, Louder AM, Eldridge EH, Lin ND, Pollock BD, Gatz JL, Grannis SJ, Vashisht R, Ghauri K, Valo G, Chakravarty AG, Lasky T, Jung M, Lovell SL, Major JM, Kabelac C, Knepper C, Leonard S, Embi PJ, Jenkinson WG, Klesh R, Garner OB, Patel A, Dahm L, Barin A, Cooper DM, Andriola T, Byington CL, Crews BO, Butte AJ, Allen J. Real-world utilization of SARS-CoV-2 serological testing in RNA positive patients across the United States. PLoS One. 2023 Feb 10;18(2):e0281365. doi: 10.1371/journal.pone.0281365. PMID: 36763574; PMCID: PMC9916659. |
| 6 | Within 10 days of COVID-19 diagnosis, describe U.S. real-world treatment patterns among patients who initiated one of four corticosteroids of interest (CSIs) — methylprednisolone (MPRED), prednisone (PRED), dexamethasone (DEX), and hydrocortisone (HC) — as used in inpatient and outpatient settings. | Describe trends and changes in treatment for hospitalized COVID-19 patients in the US, overall and stratified by COVID-19 severity (and possibly other demographics, such as facility location). | Weckstein AR, Vititoe S, Rivera DR, Bradley MC, Perez-Vilar S, Leonard S, Garry EM, Gatto NM, Rassen JA. **Temporal trends in medication utilization among hospitalized COVID-19 patients**. In: Abstracts of the 37th International Conference on Pharmacoepidemiology & Therapeutic Risk Management, Virtual, August 23, 2021. Pharmacoepidemiol Drug Saf. 2021 Aug;30 Suppl 1:351-2. doi: 10.1002/pds.5305. PMID: 34431155.  Abstracts available from: <https://onlinelibrary.wiley.com/doi/10.1002/pds.5305>.  Weckstein AR, Vititoe S, Rivera DR, Bradley MC, Perez-Vilar S, Leonard S, Garry EM, Gatto NM, Rassen JA. Temporal trends in medication utilization among hospitalized COVID-19 patients. 37th International Conference for Pharmacoepidemiology - All Access, Virtual. Poster, Aug 2021.  Perez-Vilar S, Weckstein AR, Vititoe S, Rivera DR, Bradley MC, Leonard S, Garry EM, Gatto NM, Rassen JA. **Temporal trends in medication utilization among hospitalized COVID-19 patients.** The 2021 FDA Science Forum. Science as the Foundation for Protecting and Promoting Public Health. Poster, May 26-27, 2021.  Abstract and poster available from: <https://www.fda.gov/science-research/fda-science-forum/temporal-trends-medication-utilization-among-hospitalized-covid-19-patients-united-states> |
| 7 | Describe trends in inpatient utilization and patient care over the course of the pandemic, stratified by key demographic variables (gender, race/ethnicity, region, age) and COVID-19 severity. | | Vititoe SE, Easthausen IJ, Lasky T, Chakravarty A, Bradley MC, Roe LM, Gatto NM, Weckstein AR, Garry EM. **Describing characteristics and treatment patterns of patients hospitalized with COVID-19 by race and ethnicity in a national RWD during the early months of the pandemic**. PLoS One. 2022 Sep 26;17(9):e0267815. doi: 10.1371/journal.pone.0267815. PMID: 36155644.  Full-text available from:  <https://journals.plos.org/plosone/article?id=10.1371/journal.pone.0267815>  Vititoe S, Weckstein AR, Chakravarty A, Bradley MC, Lasky T, Gatto NM, Garry EM. **Examining the Evolving Pandemic: Trends in COVID-19 Severity for Hospitalized COVID-19 Patients Over Time**. 37th International Conference for Pharmacoepidemiology - All Access, Virtual. Poster, Aug 2021.  Vititoe S, Weckstein AR, Chakravarty A, Bradley MC, Lasky T, Gatto NM, Garry EM. **Examining the Evolving Pandemic: Trends in COVID-19 Severity for Hospitalized COVID-19 Patients Over Time**. In: Abstracts of the 37th International Conference on Pharmacoepidemiology & Therapeutic Risk Management, Virtual, August 23, 2021. Pharmacoepidemiol Drug Saf. 2021 Aug;30 Suppl 1:224. doi: 10.1002/pds.5305. PMID: 34431155.  Abstracts available from: <https://onlinelibrary.wiley.com/doi/10.1002/pds.5305>. |
| 8-1 | Describe mWHO severity categorization among hospitalized COVID-19 patients (no O2; O2/NIV; IMV) using procedure-related codes related to O2 or IMV alone and the additional use of diagnoses (i.e., hypoxia, hypoxemia, and ARDS) to quantify the potential for misclassification and improve the categorization. | Develop administrative data algorithm for assessment of disease severity in hospitalized COVID-19 patients, using procedure and diagnosis codes indicative of respiratory support requirements (no O2; O2/NIV; IMV). Algorithm was refined using learnings from separate EHR dataset and assessment of potential misclassification in both EHR and administrative datasets. | Garry EM, Weckstein A, Quinto K, Lasky T, Chakravarty A, Leonard S, Vititoe S, Rassen JA, Gatto NM. **Use of an EHR to inform a claims-based algorithm to categorize inpatient COVID-19 severity**. In: Abstracts of the 37th International Conference on Pharmacoepidemiology & Therapeutic Risk Management, Virtual, August 23, 2021. Pharmacoepidemiol Drug Saf. 2021 Aug;30 Suppl 1:93. doi: 10.1002/pds.5305. PMID: 34431155.  Abstracts available from: <https://onlinelibrary.wiley.com/doi/10.1002/pds.5305>.  Garry EM, Weckstein AR, Quinto K, Lasky T, Chakravarty A, Leonard S, Vititoe S, Rassen JA, Gatto NM. **Use of an EHR to inform a claims-based algorithm to categorize inpatient COVID-19 severity.** 37th International Conference for Pharmacoepidemiology - All Access, Virtual. Podium, Aug 2021. |
| 8-2 |  | To confirm utility of the COVID-19 severity algorithm developed to categorize hospitalized patients based on respiratory support requirements (RQ8-1) by assessing whether patients categorized as having greater disease severity at admission are at higher risk of mortality. | Garry EM, Weckstein AR, Quinto K, Bradley MC, Lasky T, Chakravarty A, Leonard S, Vititoe SE, Easthausen IJ, Rassen JA, Gatto NM. **Categorization of COVID-19 severity to determine mortality risk.** Pharmacoepidemiol Drug Saf. 2022 Jul;31(7):721-728. doi: 10.1002/pds.5436. Epub 2022 May 9. PMID: 35373865; PMCID: PMC9088650  Full-text available from:  <https://pubmed.ncbi.nlm.nih.gov/35373865/>  Garry EM, Weckstein AR, Quinto K, Bradley MC, Lasky T, Leonard S, Vititoe S, Gatto NM. **Categorization of COVID-19 severity to determine mortality risk.** In: Abstracts of the 37th International Conference on Pharmacoepidemiology & Therapeutic Risk Management, Virtual, August 23, 2021. Pharmacoepidemiol Drug Saf. 2021 Aug;30 Suppl 1:91. doi: 10.1002/pds.5305. PMID: 34431155.  Abstracts available from: <https://onlinelibrary.wiley.com/doi/10.1002/pds.5305>.  Garry EM, Weckstein AR, Quinto K, Bradley MC, Lasky T, Leonard S, Vititoe S, Gatto NM. Categorization of COVID-19 severity to determine mortality risk. 37th International Conference for Pharmacoepidemiology - All Access, Virtual. Podium, Aug 2021.  Bradley MC, Garry EM, Weckstein AR, Quinto K, Lasky T, Leonard S, Vititoe S, Gatto NM. **Categorization of COVID-19 severity to determine mortality risk.** The 2021 FDA Science Forum. Science as the Foundation for Protecting and Promoting Public Health. Poster, May 26-27, 2021.  Abstract and poster available from: <https://www.fda.gov/science-research/fda-science-forum/categorization-covid-19-severity-determine-mortality-risk> |
| 9 | Within 10 days of COVID-19 diagnosis, describe U.S. real-world treatment patterns among patients who initiate oral or injectable corticosteroid therapy in an outpatient setting.^2^ | To examine utilization patterns, characteristics, and outcomes among COVID-19 patients with evidence of corticosteroid initiation in U.S. outpatient settings.^2^ | Bradley MC, Perez-Vilar S, Chillarige Y, Dong D, Martinez AI, Weckstein AR, Dal Pan GJ. **Systemic Corticosteroid Use for COVID-19 in US Outpatient Settings From April 2020 to August 2021**. *JAMA*. 2022;327(20):2015–2018. doi:10.1001/jama.2022.4877  Full-text available from:  <https://jamanetwork.com/journals/jama/fullarticle/2791078>  Bradley MC, Graham DJ, Eworuke E, Lee H, Akhtar S, Lyu H, Naik KB, Chillarige Y, Cunningham FE, Dong D, Zhang R, Cocoros NM, Cosgrove A, Martinez AI, Maro J, Weckstein AR, Baglivo A, Vititoe SE, Garry EM, Gatto NM, Kelman J, Perez-Vilar S. **Systemic corticosteroids for COVID-19 in U.S. outpatient settings.** In: ABSTRACTS of ICPE 2022, the 38th International Conference on Pharmacoepidemiology and Therapeutic Risk Management (ICPE), Copenhagen, Denmark, 26–28 August, 2022. Pharmacoepidemiol Drug Saf. 2022 Sep;31 Suppl 2:92. doi: 10.1002/pds.5518. PMID: 36148859.  Abstract available from:  <https://onlinelibrary.wiley.com/doi/10.1002/pds.5518>  Bradley MC, et al. **Systemic corticosteroids for COVID-19 in U.S. outpatient settings**. Podium presentation at: 38th International Conference for Pharmacoepidemiology & Therapeutic Risk Management; Aug 2022; Copenhagen, Denmark. |

^1^Two additional initial research questions were not pursued given the lack of a suitable data source and are therefore not listed.

^2^Aetion’s participation in these research questions stemmed from the research collaboration agreement between the U.S. Food and Drug Administration (FDA) and Aetion, Inc.; however, these research questions were part of broader projects that involved other organizations. Aetion’s progress and findings from these research questions were tracked as part of the research collaboration between the U.S. Food and Drug Administration (FDA) and Aetion, Inc.

**Article Title:** Increasing the utility of real-world data to inform public health decision making through a US-based private-public partnership: 11 lessons learned from a principled approach to rapid pandemic RWE generation

**Authors:**

Nicolle M. Gatto, PhD, MPH, Aetion, Inc., New York, NY

Elizabeth M. Garry, PhD, Aetion, Inc., New York, NY

Melanie Wang, MPH, MBA, Aetion, Inc., New York, NY

Nevine Zariffa, M. Math, NMD Group INC

Laura Roe, MMCi, Verily, San Francisco, CA

Aloka Chakravarty, PhD, U.S. Food and Drug Administration, Silver Spring, MD

Donna Rivera, PharmD, MSc, U.S. Food and Drug Administration, Silver Spring, MD

**Corresponding author:**

Nicolle M. Gatto, PhD, MPH

Aetion, Inc., 5 Pennsylvania Plaza, New York, NY 10001

[nicolle.gatto@aetion.com](mailto:nicolle.gatto@aetion.com)
