# Supplementary material for: Increasing the Utility of Real-World Data to Inform Public Health Decision Making Through a US-based Private–Public Partnership: 10 Lessons Learned from a Principled Approach to Rapid Pandemic RWE Generation
Source: Ther Innov Regul Sci. 2025 Mar 18;59(3):629–41. doi: 10.1007/s43441-025-00748-4 (PMC12018611; doi:10.1007/s43441-025-00748-4)
Supplement: Supplementary file 2 — Supplementary file2 (DOCX 16 KB) [file 43441_2025_748_MOESM2_ESM.docx]

**Table 2. Standard data characterization template**

| **#** | **Component/Question** | | **Response** |
| --- | --- | --- | --- |
| **Section 1: Overall Description** | | | |
| 1 | Data source description / any inclusion criteria applied (e.g., who is in the available data?) | |  |
| 2 | Date of most recent data cut | |  |
| 3 | Total number of unique people in the data | |  |
| 4 | Geographic Area (e.g., states covered) | |  |
| 5 | Number of data types | |  |
| 6 | Data types (e.g., insurance claims, hospital EHR, etc.) | |  |
| 7 | Data fields included in standard base dataset (i.e., without any special linkages) | |  |
| 8 | Summary of known missingness in the data overall and for key demographic fields (such as age, sex, race, state, etc.) | |  |
| 9 | Linkage potential across other datasets | |  |
| **Section 2: Timeliness** | | | |
| 10 | Dates captured | |  |
| 11 | Data lag | |  |
| 12 | Refresh rate/frequency | |  |
| **Section 3: Technical and Privacy** | | | |
| 13 | | Data model (any relevant mapping/transformation) |  |
| 14 | | De-identification/privacy protection (including tokenization measures) |  |
| 15 | | Type + level of documentation on source data |  |
| 16 | | Type + level of documentation on data transformations |  |
| 17 | | What tools, environments, and processes are required to connect this data based on data privacy and security? |  |
| 18 | | Does the data include variables that map to coding systems (e.g., ICD-9, ICD-10, CPT/HCPCS, NDC)? |  |
